# Robust Statistical Detection of Power-Law Cross-Correlation

**DOI:** 10.1038/srep27089

**Published:** 2016-06-02

**Authors:** Duncan A. J. Blythe, Vadim V. Nikulin, Klaus-Robert Müller

**Affiliations:** 1 Machine Learning Group, Berlin Institute of Technology, Berlin, Germany; 2 Bernstein Centre for Computational Neuroscience, Berlin, Germany; 3 African Institute of Mathematical Sciences, Bagamoyo, Tanzania; 4 Neurophysics Group, Department of Neurology, Charité – University Medicine Berlin, Germany; 5 Centre for Cognition and Decision Making, National Research University Higher School of Economics, Russian Federation; 6 Korea University, Seoul, South Korea

## Abstract

We show that widely used approaches in statistical physics incorrectly indicate the existence of power-law cross-correlations between financial stock market fluctuations measured over several years and the neuronal activity of the human brain lasting for only a few minutes. While such cross-correlations are nonsensical, no current methodology allows them to be reliably discarded, leaving researchers at greater risk when the spurious nature of cross-correlations is not clear from the unrelated origin of the time series and rather requires careful statistical estimation. Here we propose a theory and method (PLCC-test) which allows us to rigorously and robustly test for power-law cross-correlations, correctly detecting genuine and discarding spurious cross-correlations, thus establishing meaningful relationships between processes in complex physical systems. Our method reveals for the first time the presence of power-law cross-correlations between amplitudes of the alpha and beta frequency ranges of the human electroencephalogram.

Analysis of the relationship between distinct physical processes unfolding in parallel over time is a cornerstone in the study of complex physical systems. The standard approach to such dependencies is cross-correlation analysis. Power-law cross-correlations have generated particular interest in statistical physics^1^ due to their ubiquity^2^,^3^,^4^,^5^ and importance in critical phenomena^6^. Researchers have emphasized the need to compensate for non-stationarities which can potentially jeopardize even the most alluring interpretations of the obtained data. However, just as important as coping with non-stationarity is to distinguish true from spurious power-law cross-correlations: in the application section of this paper we show that a naïve application of current methods incorrectly indicates that the Dow Jones index over 10 years is power-law cross-correlated with the neural activity of a human subject over 10 minutes. Thus given the importance of distinguishing true from spurious power-law cross-correlation, it is surprising that there has been only one attempt^7^ to statistically test for true power-law cross-correlations in the presence of non-stationarity. Despite the advances proposed, this prior approach did not cover two important aspects, namely, generality and simultaneous testing of cross-correlation over time-scales.

Although there exist several sophisticated approaches to *quantifying* power-law cross-correlation^1^, statistical testing for power-law cross-correlation in models without non-stationarity^9^, as well as numerous non-power-law cross-correlation based approaches to studying interactions between and within complex systems (e.g.^10^,^1^1), there is no study in addition to^7^ which addresses the problem of robust statistical testing for power-law cross-correlation in the presence of non-stationarity.

In this paper we provide the first rigorous statistical approach to testing for power-law cross-correlations in the presence of non-stationarity. Our approach is based on broad assumptions—our theory depends only on a large linear semi-parametric formulation, which we show computationally may be relaxed to include non-linear processes, and our method is robust to a large class of non-stationary trends. We validate our approach in simulations and apply the framework to the Dow Jones index and electroencephalographic data to show that spurious power-law cross-correlations are correctly rejected. Finally, we show in an application to cross-frequency neural activity that novel power-law cross-correlations may be discovered using the framework.

## Theory

Let us first define power-law auto-correlation and cross-correlation. Assume processes *X*_1_(*t*) and *X*_2_(*t*) (zero mean) are long-range temporally autocorrelated (LRTC) with power-law auto-correlations:


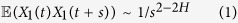



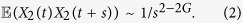


*H* and *G* are the *Hurst* exponents of *X*_1_ and *X*_2_, and are assumed to lie in the range (0.5, 1). Power-law *cross*-correlations are defined by the following relation^1^. Let *γ* ∈ (0.5, 1) and *A* and *B* be constants then:


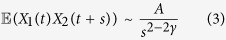



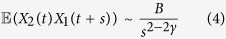


for fractional Gaussian noise processes^12^ 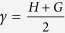; in general^13^,^1^4 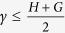.

Existing methods focus on the exponents *H*, *G* and *γ*. Since, in many applications *X*_1_ and *X*_2_ may be contaminated with non-stationary trends, detrending approaches have received significant attention. Detrended Fluctuation Analysis (DFA)^15^ estimates *H*. With 
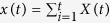
 the DFA coefficients 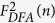, are given as the detrended variance of *x*(*t*) over windows of length *n*:


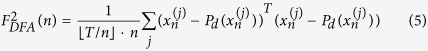


*T* is the total number of time-points recorded, 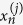 is the vector of elements of *x*(*t*) lying in the *j*^th^ time-window of length *n* and 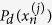 is the least squares polynomial fit of degree *d* to 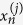. *H* is given as the least squares linear fit of log(*n*) vs. 
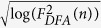
. The degree to which *X*(*t*) is power-law auto-correlated is quantified by the quality of the linear fit.

Detrended Cross-Correlation Analysis (DCCA) generalizes DFA to pairs of time-series, estimating *γ*, and quantifying scaling of cross-correlations. The DCCA coefficients are given by:


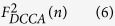



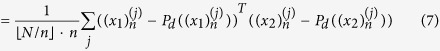



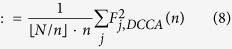


*γ* is given by the least squares linear fit of log(*n*) vs.  
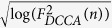
. How well the power-law cross-correlation model fits is quantified by the quality of the linear fit.

Note that alternative approaches other than DCCA for estimating *γ* exist based on averaged periodogram estimation^14^, height cross-correlation analysis^16^ and moving average approaches^17^. We focus here on DCCA due to its wide use, and as a proof of concept. A similar approach may in principle be applied to alternative estimators.

An important question left unanswered by the DCCA procedure is: how do we know that the cross-correlations over the entire range of *n* are genuine and not spurious? I.e. how do we know that the measured DCCA values and scaling relation could not have arisen from two time-series between which there are no power-law cross-correlations? It may be the case that the DCCA coefficients scale log-log linearly over a range of magnitudes but the time-series are not power-law cross-correlated, i.e. DCCA gives spurious results. If the cross-correlation are spurious then estimation of the exponent *γ* and assessing the quality of the linear fit of 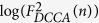 and log(*n*) make no sense.

**Figure 1:**
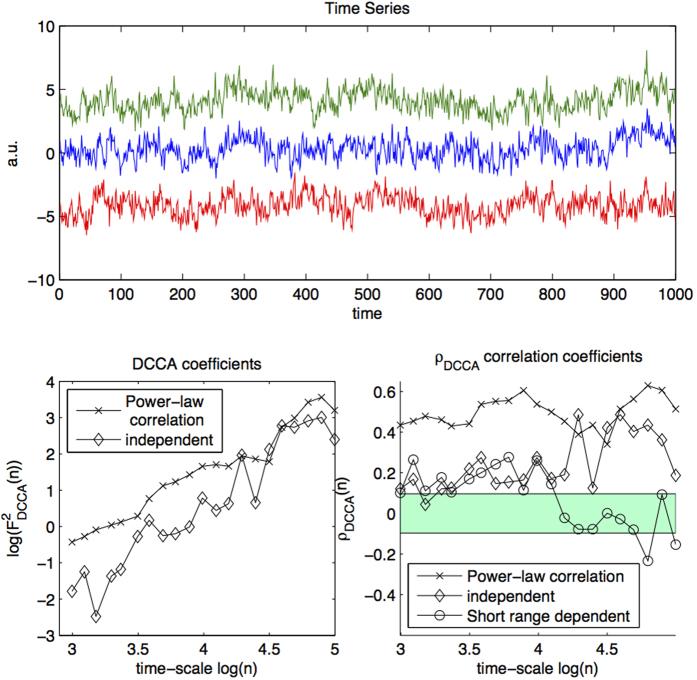
Illustration of DCCA and *ρ*_*DCCA*_. Top: Three long range temporally correlated time-series, where the top two are dependent on each other and the bottom is independent of the top two. Bottom left: DCCA coefficients in log-log coordinates. Both dependent and independent coefficients scale-log-linearly. Bottom right: *ρ*_*DCCA*_ correlation coefficients over scales. Significant correlation over the range of time-scales may be quantified exactly with the proposed method. At the 0.01 test level, all coefficients must lie on one side of the turquoise region. The test successfully discriminates between long-range dependent time-series (blue), and independent time series (green) and short-range dependent time-series (red), where correlation is only manifest at small time-scales.

On the one hand, if the coefficients 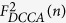 take positive and negative values over a range of time-scales clearly it does not make sense to estimate *γ* using DCCA. On the other hand, in cases in which the coefficients take only positive (or only negative) values, which scale log-linearly, it is not possible to know on this basis alone whether these cross-correlations are spurious. See Fig. 1 for an illustration: the bottom left plot displays DCCA coefficients as crosses and diamonds which are positive over the range of *n* and scale-log-linearly. Whereas the crosses are calculated from two power-law cross-correlated time-series, the diamonds are calculated from time-series which are independent: both scale log-linearly and the estimate of *γ* is larger than >0.5. This example shows that DCCA can give misleading or spurious results for time-series which are actually independent.

The problem with DCCA illustrated in the example of Fig. 1 is that 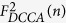 quantifies only the scaling of cross-correlations but not the absolute value of the correlations. The DCCA cross-correlation coefficient^18^ was proposed to study this distinction, which, in analogy to the Pearson correlation coefficient, is given by the DCCA coefficients normalized by the square root of the DFA coefficients:


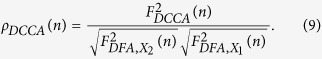


In the large *T* limit, *ρ*_*DCCA*_ quantifies the level of correlation between *X*_1_ and *X*_2_^19^; *ρ*_*DCCA*_ is 0 if *X*_1_ and *X*_2_ are independent and 1 if they are identical. The bottom-right panel of Fig. 1 illustrates *ρ*_*DCCA*_; the *ρ*_*DCCA*_ coefficients displayed as crosses are calculated from power-law cross-correlated time-series and we clearly observe that these values deviate positively from 0 across the time-scales considered.

However as with standard correlation coefficients, for finite sample sizes, *ρ*_*DCCA*_ is never 0 for independent time-series. The bottom-right panel of Fig. 1 illustrates that although the crosses, *ρ*_*DCCA*_(*n*) of power-law cross-correlated time-series, are larger in magnitude than the diamonds, *ρ*_*DCCA*_(*n*) of independent time-series, the diamonds are nevertheless greater than 0 over this range. For this reason a test was recently proposed^7^ based on surrogate data in order to calculate *p*-values for the null hypothesis of no power-law cross-correlation, addressing the question: what range can we expect the *ρ*_*DCCA*_(*n*) to take for a given *n* assuming independence?.

This approach is, however, subject to several shortcomings. Firstly the test relies on assumptions which are difficult to verify in practice: the parametric model chosen for the surrogate data and the distribution of the data should agree exactly, for *p*-values to be correct (no guarantees are given that the test is robust to differences in this distribution) and the Hurst exponents of the time-series should be known exactly.

A second and severe shortcoming of this approach is that no consideration is given as to how to incorporate the information provided by the multiple *ρ*_*DCCA*_ coefficients over time-scales: *ρ*_*DCCA*_(*n*) is considered in isolation from *ρ*_*DCCA*_(*n*′) for *n* ≠ *n*′. *ρ*_*DCCA*_(*n*) should in fact rather be considered as a multivariate vector. The bottom-left panel of Fig. 1 illustrates this important insight. The circles are *ρ*_*DCCA*_ coefficients calculated from two non-power-law cross-correlated time-series which are nevertheless short-range cross-correlated. We see that *ρ*_*DCCA*_ is large over short time-scales but small over large time-scales. This example demonstrates the need when testing for *true* power-law cross-correlations, to require large *ρ*_*DCCA*_ over a wide range of time-scales.

A question arises at this point: do we need *ρ*_*DCCA*_ values to be different from zero for all available time-scales? The answer to this question is somewhat subtle. Although it is indeed true that there are time-series models with power-law cross correlations for which asymptotic correlations vanish^13^,^1^4, in such cases we expect that *ρ*_*DCCA*_ cross-correlations vanish more slowly over a range of magnitudes than in the case of short-range cross-correlations; thus even in these cases we need to test for simultaneous cross-correlation over a range of time-scales. Indeed, considering a sizeable range of magnitudes is a pre-requisite to being able to test for power-law cross-correlation at all, since over a narrow range of magnitudes, even a non-log-log linear cross-correlation function may appear to scale log-log linearly. Exactly what range of magnitudes we consider to certify power-law cross-correlation depends on the application in question—important is that once this range of magnitudes is determined, then we need to check that there the cross-correlations across this range of magnitudes are indeed non-spurious.

Our theory allows us to calculate the asymptotic distribution of the *ρ*_*DCCA*_ coefficients, which we use in deriving a test for power-law cross-correlation. Under the assumption of linearity and independence of *X*_1_(*t*) and *X*_2_(*t*), we prove, as 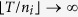, *n*_*i*_ → ∞, for *i* = 1, …, *r* (*r* number of time-scales) that the vector of *ρ*_*DCCA*_ coefficients converges in distribution to a multivariate Gaussian:


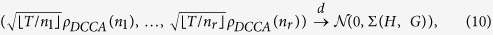


where Σ(*H*, *G*) is the covariance matrix of the limiting distribution depending only on *H* and *G* (for the detailed form of Σ(*H*, *G*) see supplement).

### Overview of Derivation

The supplement gives details of the derivation. Here we give a brief overview. The reason why standard correlation analysis is inefficient on long-range dependent data is due to the redundancy introduced by the strong autocorrelations between distant time-points^20^. The efficiency of Detrended Cross-Correlation Analysis and the Detrended Cross-Correlation Coefficient lie in the decorrelating effect of detrending on the time-varying DCCA coefficients 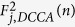. Thus we show (Lemma 1.1) that the covariance between these coefficients decays rapidly for fractional Gaussian noise:


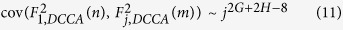


here *G*, *H* denote the Hurst exponents of the pair of time-series. Since these coefficients are short-range correlated, then we are able to derive a central limit theorem (Proposition 1.4) for 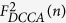, under the assumption that the analysed time-series are fractional-Gaussian noises, since 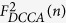 is composed as a sum of the 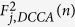.


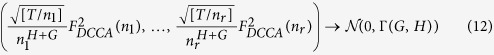


We then lever an existing central limit theorem^21^ for 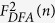 and apply the delta method to show that *ρ*_*DCCA*_(*n*) is asymptotically Gaussian with a covariance matrix which we can calculate, under the assumption that the time-series are fractional Gaussian noises (Proposition 1.5).


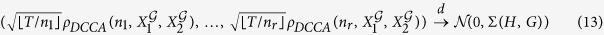


Finally we lever a known^22^ convergence result on the integrated time-series 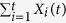 for non-Gaussian linear time-series which allows us to extend our convergence result of *ρ*_*DCCA*_(*n*) to the general linear time-series setting (Theorem 1.7); we show that since 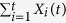 converges in distribution to 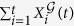, then 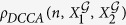 converges in distribution to *ρ*_*DCCA*_(*n*).

### Testing Procedure

Using the distribution 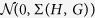, we may quantify the likelihood of observing extreme values of all *ρ*_*DCCA*_ coefficients over time scales in the absence of power-law cross-correlation, which can then be employed for rejecting or accepting the null hypothesis of no power-law cross-correlation.

Our method (denoted in the following as PLCC-test, where PLCC abbreviates power-law cross-correlation) for testing of power-law cross-correlation is based on the previous theory (available in software, see supplement). PLCC-test assesses whether significant *ρ*_*DCCA*_(*n*) values are present simultaneously across a range of time-scales. Let Σ be a covariance matrix whose diagonal elements Σ_*i*,*i*_ are equal to the maximum asympotic variance of *ρ*_*DCCA*_(*n*_*i*_) in the range *H*, *G* ∈ [0.5, 1] (max{Σ(*H*, *G*)_*i*,*i*_ | *H*, *G* ∈ (0.5, 1)}) and whose off-diagonal elements Σ_*i*,*j*_ preserve the maximum correlation between *ρ*_*DCCA*_(*n*_*i*_) and *ρ*_*DCCA*_(*n*_*j*_) for *H*, *G* ∈ (0.5, 1):


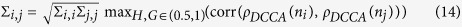


Then the probability that the *ρ*_*DCCA*_ coefficients exceed a certain level simultaneously may be bounded according to:


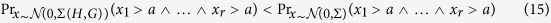


For a given test level *α* and *T*, *n*_*i*_ we use Equations (10) and (15) to find *a* such that in the asymptotic limit of *T*, *n*_*i*_:


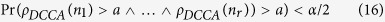


These bounds allow us to specify the rejection region, which corresponds to cases in which all DCCA coefficients display large positive (or negative) values. (see Fig. 1 bottom right with turquoise shading). In the second step of the test if the cross-correlations over the time-scales considered are non-spurious, we check that the exponent *λ* estimated using DCCA is larger than 0.5. Note that this is already guaranteed if the range of time-scales is large enough, since we have at least one of *H*, *G* > 0.5 and both *H*, *G* ≥ 0.5. This implies that *ρ*_*DCCA*_(*n*) ~* *0 for large *n*. In practice, however, we check this condition for robustness. Indeed, if 
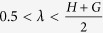
 we also have *ρ*_*DCCA*_(*n*) ~* *0. In these cases we may encounter *λ* close to 0.5.

Interestingly, PLCC-test can be extended to yield a method for calculating *p*-values for conventional cross-correlation between pairs of power-law auto-correlated time-series, potentially contaminated by non-stationary trends (see supplement, Section 3). In practice, this additional test serves to robustly assess cross-correlation in the presence of non-stationarity, no matter whether power-law or not.

## Simulations

In this section we present simulations which test the validity of our theory and method. For the generation of fractional Gaussian noise we use the implementation of^12^.

### Illustration of DCCA and the DCCA correlation coefficient

Here we present the steps necessary to reproduce Fig. 1, which illustrates the weaknesses of the naïve power-law approach and the advantages of the statistical approach.

We generate 3 time series (fractional Gaussian noise, *H* = 0.9) with length *T* = 1000 (top panel of Fig. 1). The first two are dependent with correlation *ρ* = 0.3 and the third is independent from the remainder. We then compute DCCA coefficients over the range *n* = 20, …, 150 (bottom left panel of Fig. 1). We find for this example that the coefficients between independent time-series and dependent time-series scale-log-linearly. On the other hand, we find that the *magnitude* of the *ρ*_*DCCA*_ coefficients (bottom-right of Fig. 1) reflects the degree of correlation; this observation may be completely formalized, allowing us to set the 0.01 test-level (turquoise region in Fig. 1). The *ρ*_*DCCA*_ coefficients in red correspond to independent long-range dependent time-series superimposed with correlated Gaussian white noise—correlation is visible at short but not long-time scales.

### Simulation (i)

The aim of this simulation is to test the accuracy and power of the theory and method in the case when the time-series are fractional Gaussian noises, as we asssume in the first step of our theoretical derivation.

**Figure 2:**
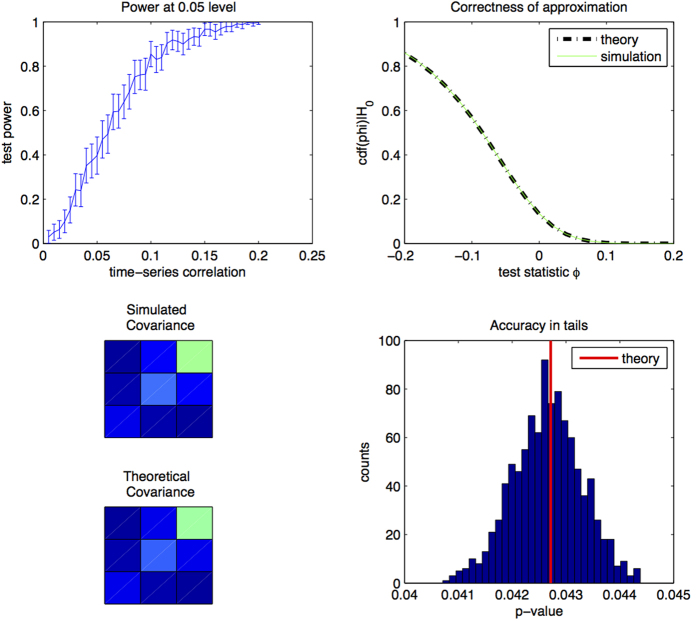
Accuracy and test-power for fractional Gaussian noise. Bottom-left: asymptotic covariance estimated by theory and comparison to covariance estimated for simulated fractional Gaussian noise; each square denotes an entry of the covariance matrices. Top-right: accuracy of theoretical cdf in comparison to simulated fractional Gaussian noise. Bottom-right: accuracy of theoretical cdf in comparison to simulated fractional Gaussian noise in the tail of the distribution. Top-left test-power as a function of correlation between components of the bivariate fractional Gaussian noise.

We simulate 100,000 pairs of independent fractional Gaussian noise of length *T* = 40,000 with *G* = 0.7, *H* = 0.8. We then measure *ρ*_*DCCA*_(*n*) settting *n* = 400, 800, 1600, 3200. The reason we consider small *n* is that the theory requires *T*/*n* → ∞. We compute quantiles of the simulated samples and compare to the asymptotic limit of Equation (10) (bottom-left, top-right and bottom-right of Fig. 2). For each value of *ρ* = 0.005, 0.01, …, 0.2 we simulate 330 pairs of correlated fractional Gaussian noises subject to a correlation of *ρ* and compute the test-power (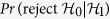). *ρ*_*DCCA*_(*n*) is calculated for *n* log-spaced values between 20 and 3000 and PLCC-test is applied at the 0.05 level. We observe good agreement with the theoretical result (in red in the bottom right and dotted-blue in the top-right of Fig. 2). The theoretical and simulated covariance of the *ρ*_*DCCA*_(*n*) coefficients are almost identical (bottom-left of Fig. 2; test-power is sufficient for usefulness of the test in practice.

### Simulation (ii)

The aim of this simulation is to test the theoretical result for non-Gaussian time-series.

**Figure 3:**
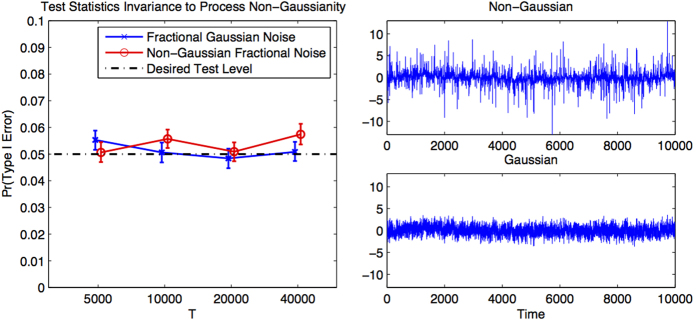
Accuracy of test for non-Gaussianity. Top right: fractional non-Gaussian noise. Bottom-right: fractional Gaussian noise subject to the same exponent as the fractional non-Gaussian (super-Gaussian) noise (above). Left: agreement of simulated samples with theory at the 0.05 test-level for both models.

We generate 10,000 pairs of respectively fractional Gaussian noise and fractional non-Gaussian noise, subject to Hurst exponents *H* = 0.7, *G* = 0.8 for each length of the time-series *T* = 5000, 10000, 20000, 40000. We then measure *ρ*_*DCCA*_(*n*) for *n* log-spaced between 60 and 500 and compare the theoretical 0.05 test-level with the 0.05 level estimated from the samples. The results are displayed in Fig. 3. The right hand panels display the sample time-series and the left hand panel displays the agreement with theory, with the 5% and 95% quantiles estimated by 1000 bootstrap iterations. We observe comparable agreement for Gaussian and non-Gaussian time-series alike.

### Simulation (iii)

The aim of this simulation is to test the theory and method in the case in which there are short-range cross-correlations but not power-law cross-correlations between the pair of times-series.

**Figure 4:**
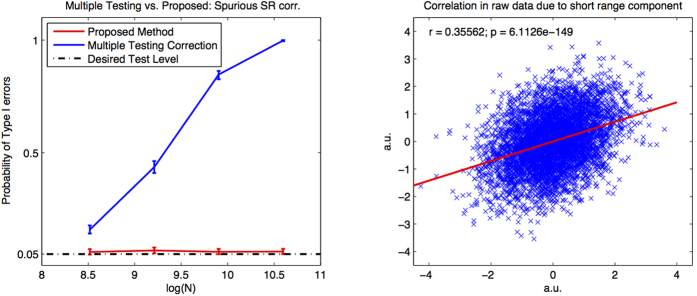
Robustness of test-statistic to short-range correlation. Right: correlation between short-range dependent time-series. Left: type I error rate estimated from pairs of simulated long range dependent time-series with short range interdependency. The desired test-level is set at 0.05. We compare the proposed method (red) with a Bonferroni multiple testing correction for tests on the individual *ρ*_*DCCA*_ coefficients (blue).

We generate 10,000 pairs of long range dependent time-series with short range interdependencies (and no long range interdependencies) by superimposing pairs of independent fractional Gaussian noises (*T* = 5000, 10000, 20000, 40000, *H* = 0) with a bivariate Gaussian white noise with standard deviation 2 and correlation *ρ* = 0.6 filtered above 2/5ths of the sampling rate with a Butterworth filter of order 4. We compute *ρ*_*DCCA*_(*n*) coefficients for *n* log-spaced between 20 and 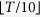 and apply PLCC-test. As a baseline we do not consider PLCC-test but apply a multiple testing correction to PLCC-test applied univariately to each of the *ρ*_*DCCA*_(*n*) coefficients. The results are displayed in Fig. 4. The left hand panel displays the agreement over iteration with the desired test-level with 5% and 95% quantiles estimated by 1000 bootstrap iterations. The right hand panel displays a sample scatter plot of the two simulated time-series against one another. While the baseline is highly sensitive to the short-range correlation, we observe that PLCC-test is robust to short-range correlation.

### Simulation (iv)

The aim of the final simulation is to test how well the theory and method generalize to non-linear time-series. Although the theory explicitly assumes linearity of the pair of processes, this condition is not strict.

We consider the non-linear Kuramoto model^23^. Which models synchronization in a population of coupled oscillators by considering only the unwrapped phase of each oscillator. The phase of each oscillator in the population, *ϕ*_*i*_, is modeled as:


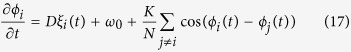


*ξ*_*i*_(*t*) is an uncorrelated noise process and cos(*ϕ*_*i*_(*t*) − *ϕ*_*j*_(*t*)) models coupling between neurons *i* and *j*. The parameter *D* controls the noise strength, *κ* controls the strength of synchrony between the oscillators *ϕ*_*i*_ and *ω*_0_ denotes the basic oscillatory frequency of the ensemble. In order to measure the joint behaviour of all *N* oscillators one considers the *mean field* of the ensemble^24^, viz.:


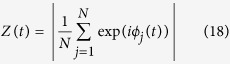


when *D* is small in comparison to *K*, the oscillators synchronize, which leads to *Z*(*t*) ≈ 1. When *D* is large in comparison to *K*, the oscillators fall out of synchronization which leads to *Z*(*t*) ≈ 0. Close to the boundary between the synchronous and asynchronous regimes, *Z*(*t*) displays fluctuations on all time-scales^25^.

**Figure 5:**
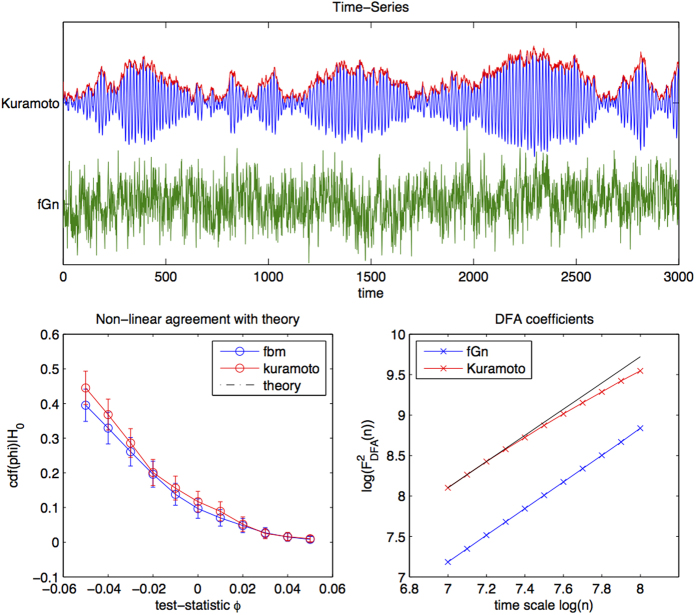
Comparison between theory, fractional Gaussian noise and Kuramoto model (non-linear, non-Gaussian). Top: mean field of Kuramoto model (above) in comparison to fractional Gaussian noise subject to the same Hurst exponent. Bottom left: empirical quantiles of *ρ*_*DCCA*_ coefficients of fractional Gaussian noise (blue) and the amplitude of the Kuramoto mean field (red) with 3 bootstrapped standard deviations as confidence. Agreement is best in the tails of the distribution. Bottom right: DFA coefficients of the amplitude of the Kuramoto mean-field and fractional Gaussian noise. We hypothesize that the slight deviation for precise scaling at higher scales explains the difference between the theory and the Kuramoto-simulated data.

We simulate pairs of independent samples from the Kuramoto model (Equation (17) setting *N* = 80, *dt* = .0.01, *K* = 0.1, *D* = 12, *ω*_0_ = 40. In the first step we determine the Hurst exponent of *Z*(*t*) (Equation (18)) 1applying DFA to log-spaced time-scales between *n* = 1000 and *n* = 3000. The reason we analyse only a narrow-range of time-scales is because the model is not strictly scale-free over a larger range of magnitudes (see bottom-right panel of Fig. 5, where we see that scaling is not exactly log-log linear), so that the theory will not provide a good approximation to the Kuramoto model when analysing a large range of *n*. Over the short-range of magnitudes studied the mean field autocorrelations are approximately log-log linear (Fig. 5, bottom-right). We then simulate pairs of fractional Gaussian noises subject to the same Hurst exponent and estimate *ρ*_*DCCA*_(*n*) coefficients for both models at the same time-scales and compare to the theory of Equation (10). The results are displayed in Fig. 5. We see that agreement holds to within bootstrap estimated confidence (1000 iterations) and improved agreement in the tails of the distribution, which is most crucial for hypothesis testing.

## Data Analysis

### Financial Data

The Dow Jones index data was downloaded from https://research.stlouisfed.org/fred2/ and extends between 2005-08-26 and 2015-08-27. Days on which no trading occurred were discarded and absolute returns were calculated as the absolute value of the difference between the index on subsequent trading days.

### EEG Data

The experimental protocol was approved by the Institutional Review Board of the Charité, Berlin. EEG recordings were obtained at rest with the subject seated comfortably in a chair in the eyes open condition. Recordings were made for three sessions of 5 minutes each, with the data set thus comprising altogether roughly 15 minutes of data. EEG data were recorded with 96 Ag/AgCl electrodes, using BrainAmp amplifiers and BrainVision Recorder software (Brain Products GmbH, Munich, Germany). The signals were recorded in the 0.016–250 Hz frequency range at a 1000 Hz sampling frequency and subsequently downsampled to 200 Hz. Outlier channels were rejected. The data were then re-referenced according to the common average. In order to obtain components with high signal to noise ratio in the alpha range, we applied Spatio-Spectral Decomposition (SSD)^26^, taking the 1st 10 components in the alpha (8–12 Hz) and beta (18–22 Hz) ranges for the subsequent analysis, each filtered forwards and backwards using Butterworth filters of order 2. Amplitude envelopes were calculated using the MATLAB implementation of the Hilbert transform/analytic signal computation (amp = abs(hilbert(x)).

### Spurious Power-Law Cross-Correlations between the Dow Jones Index and EEG Data

In the first step of our data analysis we provide a striking real-world demonstration of the possibility of apparent but spurious power-law cross-correlations between electroencephalographic (EEG) neural activity, measured over a few minutes, and the Dow Jones index (between the years 2005 and 2015, Fig. 2). The motivation of this example is *not* to reflect a real life data-analysis situation, where clearly the pairs of time-series measured would have the same length, but rather to emphasize the *dangers* of not applying proper statistical methods to empirical data. Using the standard DCCA approach one observes apparent power-law cross-correlations which are, however, discarded after statistical testing with PLCC-test. We deliberately chose brain activity of an arbitrary individual and the Dow Jones index as these are two very distinct processes which cannot relate to one another.

**Figure 6:**
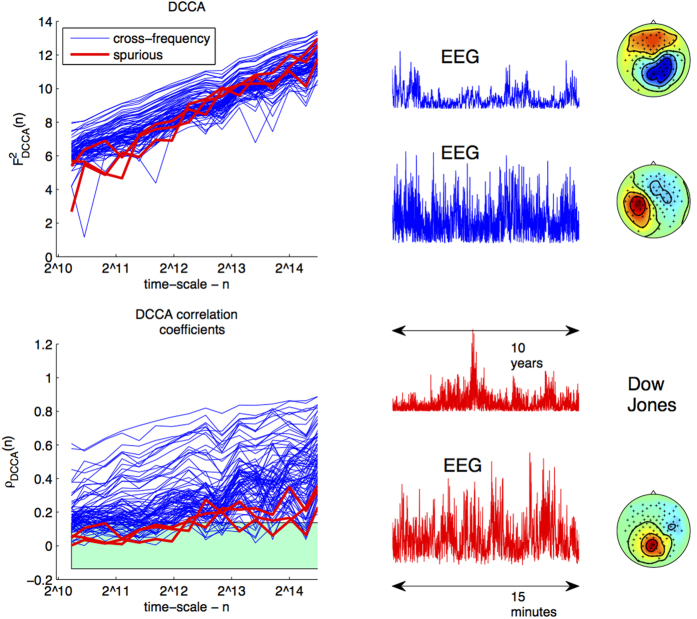
Results of analysis of EEG Data. Top left: DCCA coefficients measured between sources in the alpha range of EEG data and the Dow Jones Index (spurious correlations in red) and the beta range of the same subject (significant correlations in blue). Bottom left: DCCA correlation coefficients; significant power-law correlations must lie on one side of the turquoise region for all time-scales. Top right: time courses of significant power-law correlations. Bottom right: time courses of spurious correlations between the Dow-Jones Index and EEG. *p*-values derived using our method successfully distinguish between these cases at the *α* = 0.01 test level.

We compute DCCA coefficients and DCCA correlation coefficients (*n* ∈ [1000, 25000] between the amplitude envelopes of 10 sources extracted in the alpha range with Spatial-Spectral Decomposition (SSD)^26^ from the experimental EEG data and the absolute daily differences in the Dow Jones index. The neural times-series are down-sampled to the same *T* as the financial data. We find that of 10 correlations measured between the EEG data and the Dow Jones, 7 display both negative and positive correlations over the time-scales considered, for which the possibility of power-law correlation may be excluded. However for the three remaining pairs, DCCA analysis yields apparent, and as we know, spurious scaling behaviour (top-left of Fig. 6). These pairs each yield DCCA estimates of *γ* > 0.5, suggesting long-range dependent or even random walk behaviour (1.48, 1.32, 1.02). Only after applying PLCC-test do we find that these power-law correlations may be excluded as spurious, as expected *a priori*. In particular, application of the PLCC-test with *α* = 0.05 (Bonferroni corrected) shows that these correlations are not significant: DCCA correlation coefficients must lie outside the green region for all time-scales.

### Novel and Significant Power-Law Cross-Correlations between Alpha and Beta Range EEG

In the next step of our analysis we show that PLCC-test indeed allows for detection of novel power-law cross-correlations, by considering time-series originating from distinct brain regions and frequencies in the EEG of a given subject. It has been shown that neuronal activity in the human brain relates to critical dynamics, with persistent power-law correlations extending over multiple time scales^27^,^2^8. While many cortical areas exhibit this pattern, it is unknown whether it generalizes to a further level, whereby univariate time courses display not only power-law correlations, but also power-law *cross*-correlations. Here we provide the first evidence for such power-law cross correlations in the amplitude dynamics of distributed alpha and beta neuronal oscillations.

We follow the preprocessing steps outlined at the beginning of this section to obtain source activities using SSD. All of our subsequent analysis focuses on the amplitude envelopes (extracted by the Hilbert transform) of these source activities. Using SSD the multivariate data is decomposed as **x**(*t*) ≈ **Ws**(*t*), where 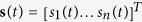. We compute separate decompositions **x**(*t*) ≈ **W**^*alpha*^**s**^*alpha*^(*t*) and **x**(*t*) ≈ **W**^*beta*^**s**^*beta*^(*t*) in the alpha (8–12 Hz) and beta (18–22 Hz) ranges respectively and take the first 10 components, after sorting the components for the signal-to-noise ratio in the corresponding frequency ranges. We then apply the Butterworth filters to the sources in the alpha and beta ranges so that they have non-overlapping frequency content. We compute DCCA coefficients and correlation coefficients (*n* ∈ [1000, 25000]) between amplitude envelopes in the alpha frequency range and 10 additional sources of the same subject in the beta range (18–22 Hz). We calculate DCCA coefficients and DCCA correlation coefficients for all pairs (n = 100) of alpha and beta sources.

Several pairs of sources display pronounced DCCA correlation coefficients. Here the testing procedure confirms that 19 of 100 pairs tested display significant power-law cross-correlations at the *α* = 0.01 test-level (Bonferroni corrected). This constitutes further evidence for the notion that when many time scales are considered jointly, the brain activity may be succinctly described through few parameters driving its coarse-grained dynamics. This finding allows us to hypothesize that such homogeneity in the behavior of neuronal oscillations across many time scales and spatial locations may be due to global modulation of cortical excitability due to varying levels of the subject’s state of arousal.

## Conclusion

While spurious power-law cross-correlations such as those observed in the brain vs. the Dow Jones index are easy to discard on the basis of simple logical considerations, detection of physically plausible cross-correlations should be based on the adequate application of robust statistical procedures.

We have proposed a versatile framework for arriving at the statistical tests for testing for power-law cross-correlation, providing an essential basis for future scientific analysis of complex multivariate dynamical systems. Our methodology should prove particularly useful in the study of noisy physical systems and cases in which weak power-law cross-correlation may be present.

Further work will aim at applying a similar approach to alternative estimators of power-law cross-correlation, such as height cross-correlation analysis^16^, detrending moving- average cross-correlation analysis^17^ and averaged periodogram estimation^14^.

## Additional Information

**How to cite this article**: Blythe, D. A. J. *et al.* Robust Statistical Detection of Power-Law Cross-Correlation. *Sci. Rep.* **6**, 27089; doi: 10.1038/srep27089 (2016).

## Supplementary Material

Supplementary Information
