# Supplementary material for: Robust Statistical Detection of Power-Law Cross-Correlation
Source: Sci Rep. 2016 Jun 2;6:27089. doi: 10.1038/srep27089 (PMC4890042; doi:10.1038/srep27089)
Supplement: Supplementary Information [file srep27089-s1.pdf]

Supplement: Robust Statistical Detection of Power-Law Cross-Correlation

Duncan A.J. Blythe, Vadim V. Nikulin, Klaus-Robert Müller

January 4, 2016

This supplement provides additional details to those provided in the main paper. Section 1 details the derivation and proof of Equation 3 of the main paper. Section 2 details the derivation of an expression for the asymptotic form of the covariance of Equation 3 of the main paper. Section 3 describes a statistical test which supplements PLCC-test. Section 4 describes the software which is available by request.

1 Theory

We assume we are given two independent processes in discrete time, $X_1(t)$ and $X_2(t)$, such that $X_1(t) = X_1^a(t) + X_1^b(t)$ and $X_2(t) = X_2^a(t) + X_2^b(t)$ where $X_1^a(t)$ and $X_2^a(t)$ are linear processes with auto-covariance functions of the form: $L_1(t)t^{2H-2}$ and $L_2(t)t^{2G-2}$. The L_i are slowly varying functions at infinity, i.e. $L_i(t)/L_i(ct) \to 1$ as $t \to \infty$ for any $c > 0$ [1, 2], and the time-series $X_i^b(t)$ are deterministic trends of a fixed polynomial order d. This formulation allows for non-Gaussianity, for confounding non-stationarities and for the presence of a high frequency component of the power-spectrum differing at those high frequencies from a power law. This semi-parametric class includes the class analysed by [3], viz. stationary Gaussian time-series with an auto covariance function proportional to $t^{2H-2}(1 + \mathcal{O}(1/t^\beta))$ since $1 + \mathcal{O}(1/t^\beta)$ is slowly varying at infinity. Moreover these assumptions rigourously formalize and generalize Equations (1) and (2) of the main paper.

We denote the integrated time-series in the lower-case: $x(t) = \sum_{i=1}^t X(t)$ and fractional Gaussian noise time series as $X^{\mathcal{G}}(t)$ and use subscripts to distinguish time-series when we consider more than one, e.g. $x_i(t), X_i(t), X_i^{\mathcal{G}}(t)$.

The main result of this section is Theorem 1.9, a central limit theorem for the vector of DCCA cross correlation coefficients; this corresponds to Equation 3 of the main paper. This is proved by first verifying the statement for the special case of fractional Gaussian noise (Proposition 1.5) and then proving that the DCCA coefficients converge to those of fractional Gaussian noise at large time-scales (Proposition 1.7).

Fractional Gaussian noise [4] is the unique Gaussian zero mean process, which is power-law auto-correlated time series with Hurst exponent H, whose integral, fractional Brownian noise $x_i^{\mathcal{G}}(t)$ has autocovariance function:

$$\mathbb{E}(x^{\mathcal{G}}(s)x^{\mathcal{G}}(t)) = \frac{\sigma^2}{2}(s^{2H} + t^{2H} - |t-s|^{2H})$$

In order to simplify notation we write $F^2_{DCCA}(n) :=_{def} F^2_{X_1,X_2}(n)$. Thus $F^2_{DFA}(n) = F^2_{X,X}(n)$.

In order that we can show that the DCCA coefficients are asymptotically normal, we first need to show that the time-varying DCCA coefficients $F^2_{j,X_1,X_2}(n)$ are sufficiently uncorrelated for application of a central limit result.

Lemma 1.1. *The covariance function:*
$cov(F^2_{1,X_1^{\mathcal{G}},X_2^{\mathcal{G}}}(n), F^2_{j,X_1^{\mathcal{G}},X_2^{\mathcal{G}}}(m))$ *has order* $j^{2G+2H-8}$ *for* $X_i^{\mathcal{G}}$ *independent fractional Gaussian noises.*

$$cov(F^2_{1,X_1^{\mathcal{G}},X_2^{\mathcal{G}}}(n), F^2_{j,X_1^{\mathcal{G}},X_2^{\mathcal{G}}}(m)) = \sigma_1^2\sigma_2^2 g(H,G) n^{H+G} m^{H+G} j^{2H+2G-8}\left(1 + \mathcal{O}(1/min(n,m)) + \mathcal{O}(1/j)\right) \quad (1)$$

Proof. Define $Q_d = I - P_d$ which is the orthogonormal projection onto the complement of the polynomials of degree d in $\mathbb{R}^n$. Let Σ_H be the univariate covariance matrix of the terms in the first and j^{th} window of

size n. Then for $d=1$:

$$\begin{aligned}
&\operatorname{cov}(F^2_{1,X_1^{\mathcal{G}},X_2^{\mathcal{G}}}(n), F^2_{j,X_1^{\mathcal{G}},X_2^{\mathcal{G}}}(m)) \\
&= \frac{2}{n^2}\operatorname{trace}(Q_d\Sigma_H Q_d \Sigma_G^\top) \\
&= \frac{2}{n^2}\operatorname{trace}(\Sigma_H\Sigma_G^\top - 2P_d\Sigma_H\Sigma_G^\top + P_d\Sigma_H P_d\Sigma_G^\top) \\
&= 2\frac{\sigma_1^2\sigma_2^2}{4} n^{2H+2G}(1+\mathcal{O}(1/n))\left(\int_0^1 (|x+j|^{2H}+y^{2H}-|x-y+j|^{2H})(|x+j|^{2G}+y^{2G}-|x-y+j|^{2G})dxdy\right. \\
&-2\int_0^1 (4-6(x+y)+12xy)(|y+j|^{2H}+z^{2H}-|z-y+j|^{2H})(|x+j|^{2G}+z^{2G}-|z-x+j|^{2G})dxdydz \\
&+\int_0^1 (4-6(x+y)+12xy)(y^{2H}+|z+j|^{2H}-|z-y+j|^{2H}) \\
&\cdot (4-6(z+w)+12zw)(x^{2G}+|w+j|^{2H}+|w-x+j|^{2G})dxdydzdw
\end{aligned}$$

After computing the Taylor expansion of the integral around $j=\infty$ in MAPLE we find it has order $j^{2G+2H-8}$. The extension to higher order detrending ($d>1$) is staightforward. When $H=G$, then we have:

$$\operatorname{cov}(F^2_{1,X_1,X_2}(n), F^2_{j,X_1,X_2}(m)) = \frac{2}{n^2}\operatorname{trace}(Q_d\Sigma_H Q_d\Sigma_H^\top)$$

But $\operatorname{trace}(Q_d\Sigma_H Q_d\Sigma_H^\top) < \operatorname{trace}(Q_1\Sigma_H Q_1\Sigma_H^\top)$, since $\operatorname{trace}(Q_d\Sigma_H Q_d\Sigma_H^\top)$ is the Froebenius norm of $Q_d\Sigma Q_d$ and Q_d projects to a subspace of the orthogonal complement of the polynomials of degree up to d. Moreover using the Cauchy-Schwarz inequality for the inner-product $\operatorname{trace}(A, B^\top)$ on matrices, the result follows for $H \neq G$. Thus, for higher degree polynomial detrending, the order of the covariance function is less than or equal to that of DCCA(1). □

Define E_j^d as the subspace of $\mathbb{R}^n$ spanned by $(\overbrace{1,1,\ldots,1}^{\times n}), ((j-1)n+1, (j-1)n+2, \ldots, jn), \ldots, ((j-1)n+1)^d, ((j-1)n+2)^d, \ldots, (jn)^d)$.

Lemma 1.2. *$E_1^d = E_j^d$ for any d (detrending degree) and $r \geq 1$.*

Proof. This may be proven directly by looking at the binomial expansion of the coefficients of the vector $(((r-1)n+1)^d, ((r-1)n+2)^d, \ldots, (rn)^d)$. □

Lemma 1.3. *$F^2_{k,X_1,X_2}(n)$ is stationary for any n.*

Proof. The proof that $F^2_{k,X_1,X_2}(n_i)$ is stationary is identical for the proof for DFA in [3] using Lemma 1.2. No modifications are necessary for polynomial trending. □

Proposition 1.4. *$(\frac{\sqrt{[T/n_1]}}{n_1^{H+G}}F^2_{X_1^{\mathcal{G}},X_2^{\mathcal{G}}}(n_1), \ldots, \frac{\sqrt{[T/n_r]}}{n_r^{H+G}}F^2_{X_1^{\mathcal{G}},X_2^{\mathcal{G}}}(n_r)) \to \mathcal{N}(0, \Gamma(G,H))$ as $[T/n_i] \to \infty$ and $n_i \to \infty$, where $\Gamma(G,H)$ is a covariance matrix which does not depend on n_i or T and where the $X_i^{\mathcal{G}}$ are independent fractional Gaussian noises.*

Proof. Let $x_i(t) = \sum_{i=1}^t X_i^{\mathcal{G}}(t)$. Define $z_i^j = \left(x_1^{(j)} - P_d\left(x_1^{(j)}\right)\right)_i$ and $w_i^j = \left(x_2^{(j)} - P_d\left(x_2^{(j)}\right)\right)_i$. Then $(z_1^j, z_2^j, \ldots, z_n^j, w_1^j, \ldots, w_n^j)$ is a stationary Gaussian vector in j. We may then use conditions on functions of a Gaussian Vector sufficient for a central limit theorem ([5], Theorem 2). It is possible to show that in the large n limit, the elements of the covariance matrix required by these conditions, which is proportional to $(I-P)\Sigma_{1,j}(1-P)$, decay faster than $1/j^2$, and thus the vector $(z_1^j, \ldots, z_n^j, w_1^j, \ldots, w_n^j)$ satisfies the conditions (since if the order is slower than this then the order of the DFA coefficients $\operatorname{cov}(F_{1,X_1^{\mathcal{G}},X_1^{\mathcal{G}}}(n), F_{j,X_1^{\mathcal{G}},X_1^{\mathcal{G}}}(n))$ is slower than j^{-4} which is impossible by an adaptation of Proposition 1.1). The generalization to the multivariate case is straightforward by way of the Cramer Wold device [6]. □

Proposition 1.5. *$(\sqrt{[T/n_1]}\rho_{DCCA}(n_1, X_1^{\mathcal{G}}, X_2^{\mathcal{G}}), \ldots, \sqrt{[T/n_r]}\rho_{DCCA}(n_r, X_1^{\mathcal{G}}, X_2^{\mathcal{G}})) \xrightarrow{d} \mathcal{N}(0, C(H,G))$ as $n_1, \ldots, n_r \to \infty$ and $[T/n_i] \to \infty$ with limiting covariance $C(H,G)$ depending only on H and G, where the $X_i^{\mathcal{G}}$ are fractional Gaussian noises.*

Proof. This then follows from the Delta method [7] levering the results of the central limit theorem for DFA [3] and DCCA (our Proposition 1.4). ☐

Theorem 1.6. *For $n \to \infty$ we have that: $L_j(n)^{-1/2}x(nt) \xrightarrow{d} \sigma B_H(nt)$ for some constant $\sigma > 0$, where $B_H(nt)$ is fractional Brownian motion with Hurst exponent H.*

Proof. See [8] for the proof. ☐

Theorem 1.7. *Assume that $Pr(sup_{s\in[0,t]}x_j(s) \geq x) \leq \frac{Cx^H}{x}$ and assume that the Hölder exponents of x_1 and x_2 are greater than or equal to the exponents of fractional Brownian motion, then as $n \to \infty$, $\lfloor T/n \rfloor \to \infty$ and $T = o\left(n^{2H+2G-\delta}\right)$ then $\frac{1}{n^{H+G}}F^2_{X_1,X_2}(n) \xrightarrow{d} \frac{\sqrt{L_1(n)L_2(n)}}{n^{H+G}}F^2_{X_1^{\mathcal{G}},X_2^{\mathcal{G}}}(n)$, where $X_i^{\mathcal{G}}$ is a fractional Gaussian noise with the same Hurst exponent as X_i.*

Proof. Our proof consists of three steps; firstly we show that the DCCA coefficients of the process given by subsampling x_1 and x_2 tend to those of fractional Brownian motion in the limit of subsampling, by using Theorem 1.6. Secondly we show that for a given ratio of subsampling to window size the DCCA coefficients of both the unsubsampled version and the subsampled version tend to each other. Thirdly we show that these imply that in the limit of window size, the DCCA coefficients tend to the law of the coefficients of fractional Gaussian noise.

Part I: We define the subsampled processes as $L_j(n)^{-1/2}X_j(nt)$. It is easy to show that the DCCA coefficients of the subsampled process tend to those of fractional Gaussian noise; this follows by Theorem 1.6 for large window sizes.

Part II: We have:

$$F^2_{1,X_1,X_2}(n) = \frac{1}{n^2}\sum_{t=1}^{n}(x_1(t) - P_d(x_1(t)))(x_2(t) - P_d(x_2(t)))$$

Let $k(x_j) = (x_j(k), x_j(2k), \ldots, x_j(n))$ and $\widehat{k}(x_j) = (\overbrace{x_j(k), \ldots, x_j(k)}^{\times k}, x_j(2k), \ldots, x_j(2k), \ldots, x_j(n))$ and let f_n^j be the least squares poly. fit of degree d to x_j and $f^j_{n/k}$ to $k(x_j)$, but with terms repeated: $f^j_{n/k} = (\overbrace{f^j_{n/k}(k), \ldots, f^j_{n/k}(k)}^{\times k}, f^j_{n/k}(2k), \ldots, f^j_{n/k}(2k), \ldots, f^j_{n/k}(n))$. Then we can use the Cauchy Schwarz inequality:

$$\begin{aligned}
& |F^2_{1,X_1,X_2}(n) - F^2_{1,k(X_1),k(X_2)}(n/k)|^2 && (2)\\
= \; & |< x_1 - f_n^1, x_2 - f_n^2 > && (3)\\
& - < \widehat{k}(x_1) - f^1_{n/k}, \widehat{k}(x_2) - f^2_{n/k} > |^2 && (4)\\
= \; & |< x_1 - f_n^1, x_2 - f_n^2 > - < \widehat{k}(x_1) - f^1_{n/k}, x_2 - f_n^2 > && (5)\\
& + < \widehat{k}(x_1) - f^1_{n/k}, x_2 - f_n^2 > && (6)\\
& - < \widehat{k}(x_1) - f^1_{n/k}, \widehat{k}(x_2) - f^2_{n/k} > |^2 && (7)\\
\leq \; & 2\Big(|< x_1 - f_n^1 - \widehat{k}(x_1) + f^1_{n/k}, x_2 - f_n^2 > |^2 && (8)\\
& + |< x_2 - f_n^2 - \widehat{k}(x_2) + f^2_{n/k}, \widehat{k}(x_1) - f^1_{n/k} > |^2\Big) && (9)\\
\leq \; & 2\Big(|x_1 - f_n^1 - \widehat{k}(x_1) + f^1_{n/k}| \times |x_2 - f_n^2| && (10)\\
& + |x_2 - f_n^2 - \widehat{k}(x_2) + f^2_{n/k}| \times |\widehat{k}(x_1) - f^1_{n/k}|\Big) && (11)\\
\leq \; & 2\Big((|x_1 - \widehat{k}(x_1)| + |f_n^1 - f^1_{n/k}|) \times |x_2 - f_n^2| && (12)\\
& + (|x_2 - \widehat{k}(x_2)| + |f_n^2 - f^2_{n/k}|) \times |\widehat{k}(x_1) - f^1_{n/k}|\Big) && (13)
\end{aligned}$$

It is possible to show, using Riemann sums, that, if f^* is the poly. of degree d which minimizes the generative mean squared error to $x(t)$ ($f^* = \min_f \int_0^1 (f(nt) - x(nt))^2$) then:

$$
\begin{aligned}
|x - f_n|^2 &= \frac{1}{n}\sum_{i=1}^{n}(x(i) - f_n)^2 && (14)\\
&= |x - f^*|^2 + \mathcal{O}(\max((x - f^*)^2)/n^2) && (15)
\end{aligned}
$$

Since least squares is a convex and differentiable optimization problem, we have that f_n and f^* are close to one another and so are $f_{n/k}$ and f^*. Therefore we can obtain bounds for $f_n - f_{n/k}$ since:

$$
|f_{n/k} - f_n|^2 \leq 2\left(|f_{n/k} - f^*|^2 + |f_n - f^*|^2\right)
$$

To obtain rates bounding the right hand expressions we need to obtain the parameter of strong convexity for the m.s.e. ($\int_0^1 (f(nt) - x(nt))^2 dt$). To do this we observe that the Hessian of the m.s.e. for the linear polynomials is calculated, letting $f(t) = a(t) + b$, by:

$$
\begin{aligned}
\partial_a\partial_a \int_0^1 (ant + b - x(nt))^2 dt &= 1/3n^2\\
\partial_a\partial_b \int_0^1 (ant + b - x(nt))^2 dt &= n\\
\partial_b\partial_a \int_0^1 (ant + b - x(nt))^2 dt &= n\\
\partial_b\partial_b \int_0^1 (ant + b - x(nt))^2 dt &= 1
\end{aligned}
$$

This implies that the smallest eigenvalue λ_1 of the Hessian matrix is a constant and greater than zero. Similarly for the polynomials of degree d we have a non-degenerate and symmetric Hessian whose smallest eigenvalue is thus greater than zero. Therefore ([9] (page 63–65)):

$$
\lambda_1\left((a_n - a^*)^2 + (b_n - b^*)^2\right) \leq \int_0^1 (x_1(nt) - f_n^1(nt))^2 dt - \int_0^1 (x_1(nt) - f^*(nt))^2 dt \qquad (16)
$$

Here we have the difference of two mean squared errors on the interval $[0, n]$. We have that with probability $1 - \frac{Cn^H}{n}$:

$$
\begin{aligned}
|f_n - f^*|^2 &= \frac{1}{n}\sum_{i}^{n}((a_n - a^*)i - (b_n - b^*))^2\\
&\leq \frac{n-1}{2}((a_n - a^*)^2 + (b_n - b^*)^2)\\
&\leq D\frac{n-1}{2}(\max((x - f^*)^2)/n^2)\\
&\leq D'n
\end{aligned}
$$

The transition from the second to the third line uses Equation (16) and Equation (15). The transition to the last line uses the assumption of the proposition.

Similarly with probability $1 - \frac{C(n/k)^H}{n/k}$:

$$
\begin{aligned}
|f_{n/k} - f^*|^2 &= \frac{1}{n/k}\sum_{i}^{n/k}((a_{n/k} - a^*)i - (b_{n/k} - b^*))^2\\
&\leq D'n/k
\end{aligned}
$$

Moreover we have that x_1 and x_2 are almost surely Hölder continuous with exponent $H - \phi$ for any $\phi < 0$ [10] and using the assumption of the proposition. Thus there exists E s.t.:

$$\begin{aligned} |x_1 - \widehat{k}(x_1)|^2 &= \frac{1}{n}\sum (x_1(i) - x_1(i - mod(i,k) + k))^2 \\ &\leq Ek^{2H-2\phi} \end{aligned}$$

More by the assumption of the proposition, with probability $1 - \frac{Cn^H}{n}$:

$$\begin{aligned} |x_1 - f_n^1| &\leq \sup|x_1| \\ &\leq n \end{aligned}$$

Equation 13 implies that with probability at least $(1 - 2\frac{n^{\max(H,G)}}{n})$:

$$\begin{aligned} &\frac{1}{n^{2H+2G}}|F^2_{1,X_1,X_2}(n) - F^2_{1,k(X_1),k(X_2)}(n/k)|^2 \\ \leq &\frac{2}{n^{2H+2G}}\Big((|x_1 - \widehat{k}(x_1)| + |f_n^1 - f_{n/k}^1|) \times |x_2 - f_n^2| + (|x_2 - \widehat{k}(x_2)| + |f_n^2 - f_{n/k}^2|) \times |\widehat{k}(x_1) - f_{n/k}^1|\Big) \\ \leq &A\frac{nk^{H-\phi} + nk^{G-\phi}}{n^{2H+2G}} \end{aligned}$$

Thus let $0 < \delta < 1$ and $k = n^\delta$. Since F^2_{j,X_1,X_2} is a stationary sequence (Lemma 1.3), then, with probability at least $(1 - 2C\frac{n^{(1-\delta)\max(H,G)}}{n^{1-\delta}})$, then:

$$\frac{1}{n^{2H+2G}}|F^2_{j,X_1,X_2}(n) - F^2_{j,\widehat{k}(X_1),\widehat{k}(X_2)}(n/k)|^2 \leq A\frac{n(n^\delta)^{H-\phi} + n(n^\delta)^{G-\phi}}{n^{2H+2G}}$$

Set $\epsilon' = 2C\frac{n^{(1-\delta)\max(H,G)}}{n^{1-\delta}})$. Then, with probability $1 - \epsilon'$:

$$\frac{1}{n^{2H+2G}}|F^2_{j,X_1,X_2}(n) - F^2_{j,\widehat{k}(X_1),\widehat{k}(X_2)}(n/k)|^2 \leq \frac{A'}{\epsilon'}\frac{n(n^\delta)^{H-\phi} + n(n^\delta)^{G-\phi}}{n^{2H+2G}}n^{(1-\delta)(\max(H,G)-1)}$$

Applying Boole's equality:

$$\begin{aligned} \Pr\Big(\frac{1}{n^{2H+2G}}|F^2_{X_1,X_2}(n) - F^2_{\widehat{k}(X_1),\widehat{k}(X_2)}(n/k)|^2 &\leq \frac{A'}{\epsilon'}\frac{n(n^\delta)^{H-\phi} + n(n^\delta)^{G-\phi}}{n^{2H+2G}}n^{(1-\delta)(\max(H,G)-1)}\Big) \quad (17)\\ &\geq 1 - [T/n]\epsilon' \quad (18) \end{aligned}$$

Set $\epsilon' = \epsilon/[T/n]$ Then:

$$\begin{aligned} \Pr\Big(\frac{1}{n^{2H+2G}}|F^2_{X_1,X_2}(n) - F^2_{\widehat{k}(X_1),\widehat{k}(X_2)}(n/k)|^2 &\leq \frac{A'}{\epsilon}[T/n]\frac{n(n^\delta)^{H-\phi} + n(n^\delta)^{G-\phi}}{n^{2H+2G}}n^{(1-\delta)(\max(H,G)-1)}\Big) \quad (19)\\ &\geq 1 - \epsilon \quad (20) \end{aligned}$$

Since:

$$\begin{aligned} &\frac{n(n^\delta)^{H-\phi} + n(n^\delta)^{G-\phi}}{n^{2H+2G+1}}n^{(1-\delta)(\max(H,G)-1)} \\ \leq &\frac{1}{n^{2H+2G-\delta}} \end{aligned}$$

Then

$$\begin{aligned} \Pr\Big(\frac{1}{n^{2H+2G}}|F^2_{X_1,X_2}(n) - F^2_{\widehat{k}(X_1),\widehat{k}(X_2)}(n/k)|^2 &\leq \frac{A'}{\epsilon}\frac{T}{n^{2H+2G-\delta}}\Big) \quad (21)\\ &\geq 1 - \epsilon \quad (22) \end{aligned}$$

Thus provided:

$$T = o\left(n^{2H+2G-\delta}\right) \tag{23}$$

then the convergence in probability holds.

PART III: to show convergence in distribution we use the Portmanteau theorem [11]. Let f be a bounded continuous function and define $S_{X_1,X_2}(n) = \frac{1}{n^{H+G}} F^2_{X_1,X_2}(n)$ and $S_{X_1^{\mathcal{G}},X_2^{\mathcal{G}}}(n) = \frac{\sqrt{L_1(n)L_2(n)}}{n^{H+G}} F^2_{X_1^{\mathcal{G}},X_2^{\mathcal{G}}}(n)$ and $k = n^{\delta}$.

$$\begin{aligned}
|\mathbb{E}(f(S_{X_1,X_2}(n)) - \mathbb{E}(f(S_{X_1^{\mathcal{G}},X_2^{\mathcal{G}}}(n)))| \leq& |\mathbb{E}(f(S_{X_1,X_2}(n)) - \mathbb{E}(f(S_{\widehat{k}(X_1),\widehat{k}(X_2)}(n/k)))| \\
&+ |\mathbb{E}(f(S_{\widehat{k}(X_1),\widehat{k}(X_2)}(n/k))) - \mathbb{E}(f(S_{\widehat{k}(X_1^{\mathcal{G}}),\widehat{k}(X_2^{\mathcal{G}})}(n/k)))| \\
&+ |\mathbb{E}(f(S_{\widehat{k}(X_1^{\mathcal{G}}),\widehat{k}(X_2^{\mathcal{G}})}(n/k))) - \mathbb{E}(f(S_{X_1^{\mathcal{G}},X_2^{\mathcal{G}}}(n)))|
\end{aligned}$$

The first and the last lines converge in virtue of Part II (conv. in prob. implies conv. in dist.) and the second line converges in virtue of Part I. □

Proposition 1.8. *Under the assumptions of Proposition 1.7, as* $n \to \infty$ *and* $[T/n] \to \infty$ *then* $\frac{1}{n^{H+G}} F^2_{X_j,X_j}(n) \xrightarrow{d} \frac{L(n)}{n^{H+G}} F^2_{X_j^{\mathcal{G}},X_j^{\mathcal{G}}}(n)$*, where the* $X_j^{\mathcal{G}}$ *is a fractional Gaussian noise with the same Hurst exponent as* X_i*.*

Proof. The proof is identical to the proof for the DCCA coefficients. □

Theorem 1.9. $(\sqrt{[T/n_1]}\rho_{DCCA}(n_1, X_1, X_2), ..., \sqrt{[T/n_r]}\rho_{DCCA}(n_r, X_1, X_2)) \xrightarrow{d} \mathcal{N}(0, C(H, G))$ *as* $n_1, \dots, n_r \to \infty$ *and* $[T/n_i] \to \infty$ *with limiting covariance* $C(H, G)$ *depending only on* H *and* G*, provided* $T = o(n^{H+G-\delta/2+1/2}\sqrt{L_1(n)L_2(n)})$

Proof. By Proposition 1.7, to apply the delta method we need that $\frac{\sqrt{\lfloor T/n \rfloor}}{\sqrt{L_1(n)L_2(n)} n^{G+H}} F^2_{X_1,X_2}(n)$ obeys the central limit theorem. Equation (21) implies that we require:

$$\frac{1}{L_1(n)L_2(n)} [T/n] \frac{T}{n^{2H+2G-\delta}} \to 0 \tag{24}$$

This holds if

$$T \quad = \quad o(n^{H+G-\delta/2+1/2}\sqrt{L_1(n)L_2(n)}) \tag{25}$$

□

2 Covariance Calculation

We now give an explicit form for the covariance of Theorem 1.9. Abbreviate Q_d to Q, where the superscripts Q^n etc. denote the window size.

$$\begin{aligned}
\mathrm{cov}(F_{j',X_1,X_2}(n), F_{j,X_1,X_2}(m)) &= \frac{1}{nm}\mathrm{cov}((Q^n X_1^{(j')})^\top Q^n X_2^{(j')}, (Q^m X_1^{(j)})^\top Q^m X_2^{(j)}) \\
&= \frac{1}{nm}\mathbb{E}((Q^n X_1^{(j')})^\top Q^n X_2^{(j')} (Q^m X_1^{(j)})^\top Q^m X_2^{(j)}) - \mathbb{E}((Q^n X_1^{(j')})^\top Q^n X_2^{(j')})\mathbb{E}((Q^m X_1^{(j)})^\top Q^m X_2^{(j)}) \\
&= \frac{1}{nm}\sum_{i,k}\mathbb{E}(Q^n X_{1,i}^{(j')} Q^n X_{2,i}^{(j')} Q^m X_{1,k}^{j} Q^m X_{2,k}^{(j)}) - \mathbb{E}(Q^n X_{1,i}^{(j')} Q^n X_{2,i}^{(j')})\mathbb{E}(Q^m X_{1,k}^{(j)} Q^m X_{2,k}^{(j)}) \\
&= \frac{1}{nm}\sum_{i,k}\mathbb{E}(Q^n X_{1,i}^{(j')} Q^m X_{1,k}^{(j)})\mathbb{E}(Q^n X_{2,i}^{(j')} Q^m X_{2,k}^{(j)}) \\
&= \frac{1}{nm}\mathrm{trace}(Q^n \Sigma^{j',j}_{X_1,X_1} Q^m (Q^n \Sigma^{j',j}_{X_2,X_2} Q^m)^\top) \\
&= \frac{1}{nm}\mathrm{trace}(Q^n \Sigma^{j',j}_{X_1,X_1} Q^m (\Sigma^{j',j}_{X_2,X_2})^\top)
\end{aligned}$$

Following similar steps we find that

$$\mathrm{cov}(F_{j',X_1,X_1}(n), F_{j,X_1,X_1}(m)) = \frac{2}{n^2}\mathrm{trace}(Q^n \Sigma^{j',j}_{X_1,X_1} Q^m (\Sigma^{j',j}_{X_1,X_1})^\top)$$

Moreover we find that:

$$\begin{aligned}\mathrm{cov}(F_{j',X_1,X_2}(n), F_{j,X_1,X_1}(m)) &\propto \mathrm{trace}(Q^n \Sigma_{X_1}^{j',j} Q^m (\Sigma_{X_1,X_2}^{j',j})^\top) \\ &= 0\end{aligned}$$

and:

$$\mathrm{cov}(F_{j',X_1,X_1}(n), F_{j,X_2,X_2}(m)) = 0$$

Then:

$$\mathrm{cov}(F^2_{X_1,X_2}(n), F^2_{X_1,X_2}(m)) = \frac{1}{\lfloor N/n \rfloor \lfloor N/m \rfloor} \sum_{j',j} \mathrm{cov}(F^2_{j',X_1,X_2}(n), F^2_{j,X_1,X_2}(m)) \tag{26}$$

$$\mathrm{cov}(F^2_{X_1,X_1}(n), F^2_{X_1,X_1}(m)) = \frac{1}{\lfloor N/n \rfloor \lfloor N/m \rfloor} \sum_{j',j} \mathrm{cov}(F^2_{j',X_1,X_1}(n), F^2_{j,X_1,X_1}(m)) \tag{27}$$

$$\mathrm{cov}(F^2_{X_1,X_1}(n), F^2_{X_1,X_2}(m)) = \mathrm{cov}(F^2_{X_1,X_1}(n), F^2_{X_2,X_2}(m)) \tag{28}$$

$$= 0 \tag{29}$$

To apply the delta method we consider the vector:

$$Z = (F^2_{X_1,X_2}(n_1), F^2_{X_1,X_1}(n_1), F^2_{X_2,X_2}(n_1), \dots, F^2_{X_1,X_2}(n_r), F^2_{X_1,X_1}(n_r), F^2_{X_2,X_2}(n_r))$$

which has covariance C', whose entries are given by Equations (26) to (29) and mean given by [3]:

$$\mu = (0, f(H)n^{2H}, f(G)n^{2G}, \dots)$$

where $f(H) = \frac{(1-H)}{(2+2H)(H+1)(H+2)}$ [3]. The covariance of the DCCA correlation coefficients is given by the covariance of $h(Z) = (\frac{Z_1}{\sqrt{Z_2 Z_3}}, \dots, \frac{Z_{3r-2}}{\sqrt{Z_{3r-1}Z_{3r}}})$, which is given by:

$$\frac{\partial h(Z)}{\partial Z} \times C' \times \left(\frac{\partial h(Z)}{\partial Z}\right)^\top$$

Thus since,

$$\begin{aligned}\frac{\partial}{\partial Z_1} h(Z)_1 &= \frac{1}{\sqrt{Z_2 Z_3}} \\ \frac{\partial}{\partial Z_2} h(Z)_1 &= -\frac{Z_1}{(Z_2 Z_3)^{3/2}} \\ \frac{\partial}{\partial Z_3} h(Z)_1 &= -\frac{Z_1}{(Z_2 Z_3)^{3/2}}\end{aligned}$$

And since the mean under the null hypothesis of $h(Z)$ is 0 (under $\mathcal{H}_0$), so that the second two derivative terms are zero, then the delta method implies we have:

$$\mathrm{cov}(\rho_{DCCA}(n_i), \rho_{DCCA}(n_j)) = (\nabla h(\mu)^\top C' \nabla h(\mu))_{i,j} \tag{30}$$

$$= \frac{C'_{3(i-1)+1,3(j-1)+1}}{\sqrt{\mathbb{E}(Z_{3(i-1)+2})\mathbb{E}(Z_{3(i-1)+2})}\sqrt{\mathbb{E}(Z_{3(j-1)+2})\mathbb{E}(Z_{3(j-1)+2})}} \tag{31}$$

Thus

$$\Sigma(H,G)_{i,j} = \sqrt{\lfloor T/n_i \rfloor \lfloor T/n_j \rfloor} \times \mathrm{cov}(\rho_{DCCA}(n_i), \rho_{DCCA}(n_j)) \tag{32}$$

3 Test 2: Cross-Correlation between Power-Law Autocorrelated Processes

The second test is a test for cross-correlation, which is not necessarily power-law cross-correlation. Its novelty lies in the fact that it is valid for general power-law auto-correlated time-series, potentially contaminated with non-stationary trends, and maximizes the test power among coefficients derived from our theory.

We find a positive measure $\mathbf{w} \in \mathbb{R}^r$ so that $\mathbf{w}^\top \rho_{DCCA}(\mathbf{n})$ has maximum Σ variance.

$$\mathbf{w} = \text{argmax}_{\mathbf{u} \in \mathbb{R}^r : u_i, \geq 0 \sum_{i=1}^r u = 1} \mathbf{u}^\top \Sigma \mathbf{u}$$

The choice of Σ ensures that:

$$\mathbf{w}^\top \Sigma(H, G) \mathbf{w} < \mathbf{w}^\top \Sigma \mathbf{w}$$

We proceed as for Test 1 (in the main paper), finding a such that in the large N, n_i limit:

$$\Pr(\mathbf{w}^\top \rho_{DCCA}(\mathbf{n}) < a) < \alpha \tag{33}$$

4 Software

`rho_DCCA.m` calculates ρ_{DCCA} correlation coefficients over scales for arbitrary degree detrending.
`test_scaling_region` calculates p-values for the null-hypothesis of no power-law correlation.
`test_minimum_variance` calculates the p-values for the correlation coefficient maximizing test-power over a specified range of scales.

References

[1] J. Karamata, "Sur un mode de croissance réguliere des fonctions," *Bull. Soc. Math.*, vol. 62, pp. 55–62, 1933.

[2] J. Lamperti, "Semi-stable stochastic processes," *Transactions of the American Mathematical Society*, vol. 104, no. 1, pp. 62–78, 1962.

[3] J.-M. Bardet and I. Kammoun, "Asymptotic properties of the detrended fluctuation analysis of long range dependent processes," *IEEE Transactions on information theory*, vol. 54, pp. 2041 – 2052, September 2007.

[4] B. B. Mandelbrot and J. W. Van Ness, "Fractional Brownian motions, fractional noises and applications," *SIAM review*, vol. 10, no. 4, pp. 422–437, 1968.

[5] M. Arcones, "Limit theorems for nonlinear functionals of a stationary Gaussian sequence of vectors," *The Annals of Probability*, vol. 22, no. 4, pp. 2242–2274, 1994.

[6] H. Cramér and H. Wold, "Some theorems on distribution functions," *Journal of the London Mathematical Society*, vol. 1, no. 4, pp. 290–294, 1936.

[7] G. Casella and R. L. Berger, *Statistical inference*, vol. 2. Duxbury Pacific Grove, CA, 2002.

[8] M. S. Taqqu, "Weak convergence to fractional Brownian motion and to the Rosenblatt process," *Probability Theory and Related Fields*, vol. 31, no. 4, pp. 287–302, 1975.

[9] Y. Nesterov and I. E. Nesterov, *Introductory lectures on convex optimization: A basic course*, vol. 87. Springer, 2004.

[10] L. Decreusefond and A. S. Üstünel, "Stochastic analysis of the fractional brownian motion," *Potential analysis*, vol. 10, no. 2, pp. 177–214, 1999.

[11] A. Klenke, *Probability theory: a comprehensive course.* Springer, 2008.

[12] P.-O. Amblard, J.-F. Coeurjolly, F. Lavancier, and A. Philippe, "Basic properties of the multivariate fractional Brownian motion," in *Séminaires et Congres*, vol. 28, pp. 65–87, 2013.

[13] R. S. Blum, "A simple model for fractional non-gaussian processes," in *Time-Frequency and Time-Scale Analysis, 1994., Proceedings of the IEEE-SP International Symposium on*, pp. 444–447, IEEE, 1994.

[14] Y. Kuramoto, *Chemical oscillations, waves, and turbulence.* Courier Corporation, 2003.

[15] A. Pikovsky, M. Rosenblum, and J. Kurths, "A universal concept in nonlinear sciences," *Self*, vol. 2, p. 3, 2001.

[16] H. Daido, "Intrinsic fluctuations and a phase transition in a class of large populations of interacting oscillators," *Journal of Statistical Physics*, vol. 60, no. 5-6, pp. 753–800, 1990.

[17] V. V. Nikulin, G. Nolte, and G. Curio, "A novel method for reliable and fast extraction of neuronal EEG/MEG oscillations on the basis of spatio-spectral decomposition," *Neuroimage*, vol. 55, no. 4, pp. 1528–1535, 2011.
